# Impact of intravenous alteplase on sub-angiographic emboli in high-resolution diffusion-weighted imaging following successful thrombectomy

**DOI:** 10.1007/s00330-021-07980-0

**Published:** 2021-05-08

**Authors:** Gabriel Broocks, Lukas Meyer, Reza Kabiri, Helge C. Kniep, Rosalie McDonough, Matthias Bechstein, Noel van Horn, Thomas Lindner, Jan Sedlacik, Bastian Cheng, Götz Thomalla, Gerhard Schön, Jens Fiehler, Uta Hanning, Michael H. Schönfeld

**Affiliations:** 1 Department of Diagnostic and Interventional Neuroradiology, University Medical Center Hamburg-Eppendorf, Martinistrasse 52, 20246 Hamburg, Germany; 2 Centre for the Developing Brain & Biomedical Engineering Department, School of Biomedical Engineering & Imaging Sciences, King’s College London, London, UK; 3 Department of Neurology, University Medical Center Hamburg-Eppendorf, Hamburg, Germany; 4 Institute of Epidemiology and Medical Biometry, University Medical Center Hamburg-Eppendorf, Hamburg, Germany; 5 Department of Neuroradiology, University Köln, Köln, Germany

**Keywords:** Stroke, Thrombectomy, Magnetic resonance imaging, Ischemia, Infarction

## Abstract

**Objective:**

Thrombus microfragmentation causing peripheral emboli (PE) during mechanical thrombectomy (MT) may modulate treatment effects, even in cases with successful reperfusion. This study aims to investigate whether intravenous alteplase is of potential benefit in reducing PE after successful MT.

**Methods:**

Patients from a prospective study treated at a tertiary care stroke center between 08/2017 and 12/2019 were analyzed. The main inclusion criterion was successful reperfusion after MT (defined as expanded thrombolysis in cerebral infarction (eTICI) scale ≥ 2b50) of large vessel occlusion anterior circulation stroke. All patients received a high-resolution diffusion-weighted imaging (DWI) follow-up 24 h after MT for PE detection. Patients were grouped as “direct MT” (no alteplase) or as MT plus additional intravenous alteplase. The number and volume of ischemic core lesions and PE were then quantified and analyzed.

**Results:**

Fifty-six patients were prospectively enrolled. Additional intravenous alteplase was administered in 46.3% (26/56). There were no statistically significant differences of PE compared by groups of direct MT and additional intravenous alteplase administration regarding mean numbers (12.1, 95% CI 8.6–15.5 vs. 11.1, 95% CI 7.0–15.1; *p* = 0.701), and median volume (0.70 mL, IQR 0.21–1.55 vs. 0.39 mL, IQR 0.10–1.62; *p* = 0.554). In uni- and multivariable linear regression analysis, higher eTICI scores were significantly associated with reduced PE, while the administration of alteplase was neither associated with numbers nor volume of peripheral emboli. Additional alteplase did not alter reperfusion success.

**Conclusions:**

Intravenous alteplase neither affects the number nor volume of sub-angiographic DWI-PE after successful endovascular reperfusion. In the light of currently running randomized trials, further studies are warranted to validate these findings.

**Key Points:**

*• Thrombus microfragmentation during endovascular stroke treatment may cause peripheral emboli that are only detectable on diffusion-weighted imaging and may directly compromise treatment effects.*

*• In this prospective study, the application of intravenous alteplase did not influence the occurrence of peripheral emboli detected on high-resolution diffusion-weighted imaging.*

*• A higher degree of recanalization was associated with a reduced number and volume of peripheral emboli and better functional outcome, while contrariwise, peripheral emboli did not modify the effect of recanalization on modified Rankin Scale scores at day 90.*

**Supplementary Information:**

The online version contains supplementary material available at 10.1007/s00330-021-07980-0.

## Introduction

Mechanical thrombectomy (MT) is of benefit to patients with acute ischemic stroke and large vessel occlusion (LVO) in the anterior circulation [1]. Although intravenous (IV) thrombolysis with alteplase, a recombinant tissue plasminogen activator, is the standard medical treatment for acute ischemic stroke patients, it is still ambiguous whether its application prior MT improves functional outcome in patients with LVO compared to direct MT [2]. It is well established that the key for improving functional outcome is successful revascularization, as demonstrated in multiple trials [3, 4]. IV lysis alone, however, rarely leads to sufficient recanalization in LVO stroke and might increase the risk of secondary hemorrhage [5, 6]. In the light of the recently published randomized controlled trial “DIRECT MT” [7] that observed non-inferiority of direct MT compared to MT with bridging IV lysis, it becomes even more relevant to understand the effect of alteplase on ischemic lesion pathophysiology [7, 8].

Potential advantages of IV lysis may include improved microvascular reperfusion and lysis of periperhal emboli caused by thrombus (micro)fragmentation during MT [9]. Recently, it has been reported that the so-called sub-angiograpic peripheral emboli (PE) can be detected on high-resolution diffusion-weighted imaging (DWI), including cases in which complete angiographic reperfusion was achieved [10]. Accordingly, bridging IV alteplase prior to MT might mediate the occurrence of PE; however, this hypothesis has yet to be investigated. This prospective cohort study analyzes the impact of IV alteplase on PE after successful MT. We hypothesized that the administration of bridging alteplase prior to MT is associated with smaller numbers and/or volumes of postinterventional PE detectable on high-resolution DWI compared to direct MT.

## Material and methods

### Study population

Patient data was collected in a prospective non-randomized cohort study treated at a tertiary care stroke center between 08/2017 and 12/2019. Inclusion criteria were defined as (1) CT-angiography confirmed proximal LVO anterior circulation stroke without signs of dissection, (2) treated with MT, leading to (3) successful reperfusion defined as an expanded Thrombolysis in Cerebral Infarction (eTICI) score ≥ 2b50 [11]. The study was approved by the local ethics committee (Ethics Committee of the Physician Board Hamburg; approval number WF 019/19) and all patients gave written informed consent (or by authorized family members/ legal guardians) according to the guidelines of the local ethics committee. This study was conducted in accordance with the Declaration of Helsinki and the STROBE guidelines. The standard imaging modality at baseline was computed tomography, with the intention not to delay endovascular treatment, in line with current guideline recommendations [12].

### Procedure

If eligible, all patients admitted up to 4.5 h after symptom onset received bridging IV alteplase (0.9 mg per kg body weight) prior to MT according to German guidelines [13]. Procedures were performed under general anesthesia or conscious sedation. Choice of access material and thrombectomy devices was at the discretion of the acting neuro-interventionalist in charge.

### Clinical data

Baseline demographic data, National Institutes of Health Stroke Scale (NIHSS) score on admission and discharge, and modified Rankin scale (mRS) scores at 90-days follow-up were documented and analyzed.

### Follow-up imaging and analysis

**Fig. 1:**
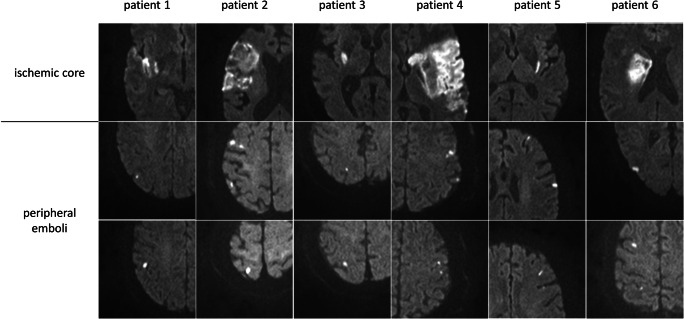
Example of six patients with ischemic cores (upper row) and peripheral emboli (lower rows) on the trace images of the high-resolution DWI

Postinterventional digital subtraction angiography (DSA) imaging was evaluated using the eTICI score, as proposed by Liebeskind et al [14]. Follow-up MRI was performed on a 1.5-T MRI scanner (Siemens Magnetom Avanto) within 24 h following intervention and included axial whole-brain high-resolution DWI using single-shot, multi-slice, spin-echo, echo-planar imaging sequences with diffusion gradients in three orthogonal directions and the following parameters: TR/TE 13,000/90 ms, *b* values of 0, 500, and 1000s/mm^2^, matrix 192×192, no gap, field of view 240, slice thickness 2 mm, acquisition time of 457 s. The spatial resolution in the 3D volume was increased, in addition to increasing both the TR and TE and the number of excitations substantially to improve the signal-to-noise ratio and to reduce the occurrence of sequence-related artifacts (see Supplemental Figure III). PE were detected as visually evident focal diffusion restrictions with a high signal on the trace images with a *b* value of 1000 and a reduced ADC-value on the ADC maps distal to a continuous core high-resolution DWI lesion (Fig. 1). DWI lesions were segmented semi-automatically with the Analyze 11.0 software package (AnalyzeDirect, Inc.) using seed-growing algorithms. Subsequently, the total PE volume in milliliters was derived automatically in Analyze using the sampling option. DSA and MRI analyses were performed independently by two readers who did not participate in the endovascular therapy. The imaging analysis was performed blinded to any clinical data including treatment. Discrepancies between readers were resolved by consensus in a joint side-by-side comparison of segmented lesion maps and joint selection and manual selection of the appropriate lesion maps.

### Statistical analysis

Standard descriptive statistics were applied for all data. Patients who received IV lysis were compared to patients without IV lysis (Table 1). Wilcoxon signed rank test was performed to compare continuous variables, as appropriate. The chi-square test and Fisher’s exact test were used to compare categorial variables. A subgroup analysis for patients with a time from symptom onset to reperfusion ≤ 6h was performed. Furthermore, the association of IV alteplase and number/volume of peripheral emboli was analyzed using univariable and multivariable linear regression analysis with eTICI, ASPECTS, time from initiation of IV alteplase to reperfusion, and age as independent parameter using backwards selection.

**Table 1:** Overview of patients’ baseline characteristics, and comparison grouped by IV alteplase administration

Baseline characteristics	All patients (*n* = 56)	IV alteplase (*n* = 26)	No IV alteplase (*n* = 30)	*p* value
Age in years, median (IQR)	71.5 (63–81)	71.5 (66.75–81)	71 (62–82.25)	0.837
Female sex, *n* (%)	26 (46.4)	10 (38.5)	16 (53.3)	0.266
Atrial fibrillation, *n* (%)	24 (42.9)	12 (46.2)	20 (66.7)	0.122
Admission NIHSS, median (IQR)	13.5 (10–16.75)	12.5 (10–17)	14 (9.75–16.25)	0.921
Occlusion site, *n* (%)				
Tandem occlusion	9 (16.1)	3 (5.4)	6 (10.7)	0.481
Terminal ICA	12 (21.4)	6 (10.7)	6 (10.7)	0.521
MCA	43 (76.8)	19 (33.9)	24 (42.9)	
ACA	1 (1.8)	1 (1.8)	0	
ASPECTS, median (IQR)	8 (6–9)	9 (6–10)	8 (6–9)	0.073

The two-way analysis of variance (ANOVA) was performed to investigate the effect of alteplase on peripheral emboli according to the reperfusion grade (dependent variable: number of PE and volume of PE; with reperfusion grade according to eTICI, and application of alteplase as independent variables; see supplemental content).

Equivalence tests were performed to further investigate whether there is a significant difference comparing the number and volume of peripheral emboli in patients with versus without IV alteplase (see supplemental content).

Finally, the impact of intravenous alteplase on functional outcome, as well as the association of PE and functional outcome, was investigated using univariable and multivariable ordinal regression analysis.

Statistics were calculated using SPSS 24.0 (IBM SPSS Statistics for Windows: IBM Corp.) and StataSE (StataCorp). *p* values < 0.05 were considered statistically significant.

## Results

### Patient population

Fifty-six patients met the inclusion criteria and were prospectively enrolled and analyzed. The median age was 71.5 years (IQR 63–81) and 46.4% (26/56) were female. Median ASPECTS was 8 (6–9) and median NIHSS was 13.5 (10–16.75) on admission. A total of 46.4% (26/56) of all patients received IV alteplase prior to MT. The mean time from symptom onset to imaging was 2.8 h (SD: 1.0) in patients with bridging IV alteplase, which was shorter compared to patients with direct MT (mean time 5.4 h, SD: 5.0). The mean time from symptom onset to reperfusion was 6.5 h (SD: 3.0 h). Table 1 provides an overview of patients’ baseline characteristics, and comparison grouped by IV alteplase administration. Time from symptom onset to reperfusion was significantly longer (*p* = 0.002) in the group of direct MT (370 min, IQR 249–574.5 vs. 255 min, IQR 201–338.5). There were no significant differences in the distribution of reperfusion grades (7/56 eTICI 2b50; 17/56 eTICI 2b67; 12/56 eTICI 2c; 20/56 eTICI 3) between the two groups (*p* = 0.740).

Three out of 56 patients (5.3%) showed secondary intracranial hemorrhage on follow-up imaging (as stated in the MRI report, which is compiled by an experienced board-certified neuroradiologist and verified by a further experienced attending neuroradiologist). None of these patients fulfilled criteria of a symptomatic hemorrhage (defined as NIHSS increase > 4, NIHSS increase > 2 points in one category, clinical deterioration leading to intubation, hemicraniectomy, or other major medical/surgical intervention).

### Impact of intravenous alteplase on peripheral emboli

**Fig. 2:**
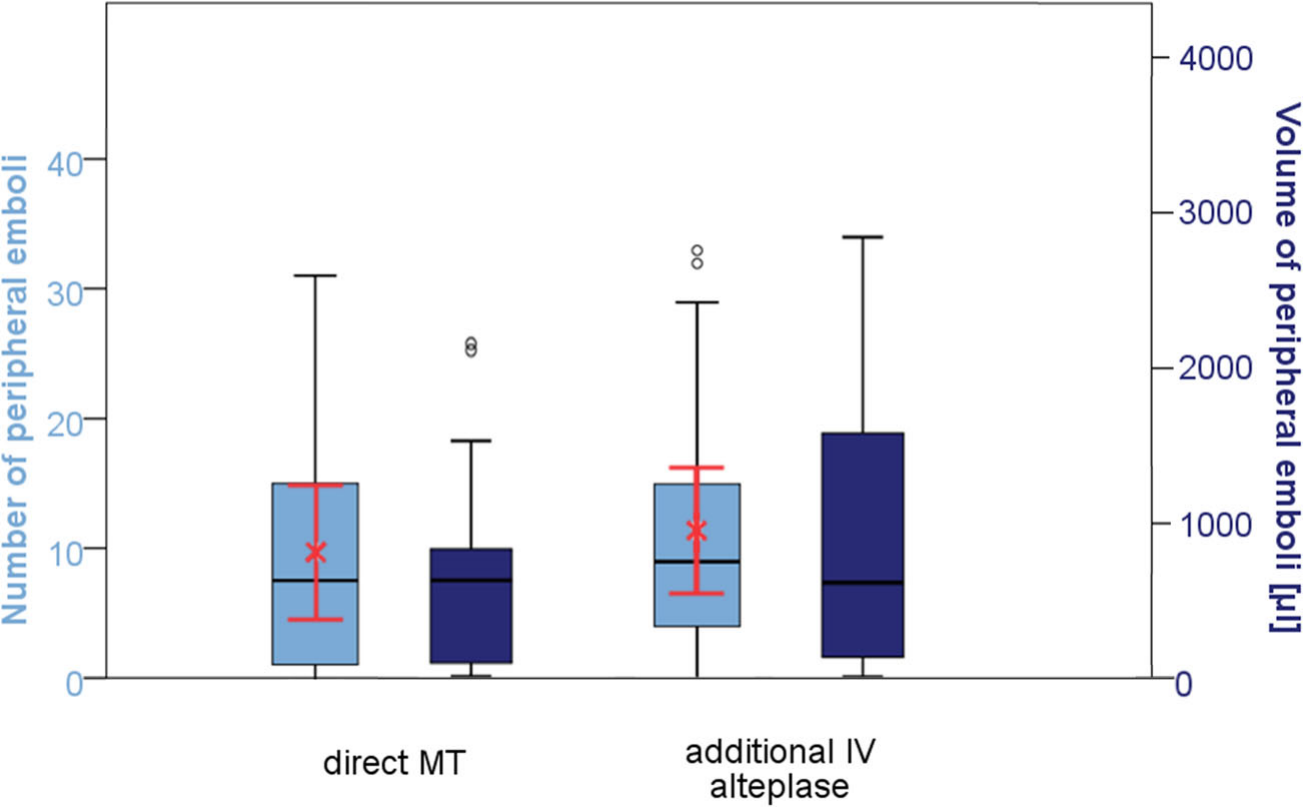
Box plots and means (red crosses) with 95% confidence interval (red whiskers) of numbers (light blue) and volume (dark blue) of peripheral emboli compared by patients treated with (*n* = 21) and without (*n* = 14) additional intravenous alteplase in patients with a time from symptom onset to reperfusion ≤ 6 h

The mean number of PE was 11.6 (95% CI 9.1–14.2) and did not differ significantly between those with direct MT and those with additional IV alteplase numbers (12.1, 95% CI 8.6–15.5 vs. 11.1 95% CI 7.0–15.1; *p* = 0.701, respectively). In all patients, the median volume of the core infarct was 19.33mL (IQR 6.08–54.51) and the median volume of all PE was 0.62mL (IQR 0.12–1.57). A volumetric-based comparison of the study groups showed no significant differences in all patients or the subgroup of patients with a time from symptom onset to reperfusion ≤ 6h (Fig. 2). In the cohort of patients receiving additional alteplase, no significant relationship between the time from initiation of IV alteplase to reperfusion and number (*p* = 0.833) or volume of PE (*p* = 0.871) was observed.

In univariable linear regression analysis, IV alteplase was not significantly associated number of PE (*ß*: − 0.99, 95% CI: − 6.1 to 4.2; *p* = 0.7) or volume of PE (*ß*: − 130.9, 95% CI: − 957.3 to 695.5; *p* = 0.8). In multivariable linear regression analysis, IV alteplase was neither associated with number (*ß*: − 1.6, SD: 2.1; *p* = 0.45) nor volume of PE (*ß*: − 133.5, SD: 422.7; *p* = 0.75). The degree of reperfusion indicated by eTICI was the only remaining variable to be significantly and independently associated with the number of PE (*ß*: − 5.6, SD: 1.0; *p* < 0.0001), and volume of PE (*ß*: − 371.8, SD: 187.5; *p* = 0.04), adjusted for ASPECTS, age, and application of IV alteplase. Table 2 provides an overview and comparison of procedural and functional outcome together with the results of the imaging analysis according to IV alteplase administration.

**Table 2:** Comparison of procedural and functional outcome, and imaging analysis between patients treated with and without intravenous alteplase

Procedural and functional outcome	All patients (*n* = 56)	IV alteplase (*n* = 26)	No IV alteplase (*n* = 30)	*p* value
eTICI (2b50/2b67/2c/3)	7/17/12/20	4/8/4/10	3/9/8/10	0.740
Time symptom onset to reperfusion in min, median (IQR) [*n* = 54]	281.5 (230–465.5)	255 (201–338.5)	370 (249–574.5)	0.022*
Time IV alteplase administration to reperfusion in min, median (IQR)	–	148 (104.75–205.25)	–	-
mRS 90 days, median (IQR)	2 (0–3) [*n* = 53]	1.5 (0–3) [*n* = 26]	2 (1–4) [*n* = 27]	0.152
Imaging analysis				
Number of peripheral emboli, median (mean; 95% CI)	11.6 (9.1–14.2)	11.1 (7.0–15.1)	12.1 (8.6–15.5)	0.701
Time from symptom onset to reperfusion ≤ 6h	10.6 (7.2–14.0) [*n* = 35]	11.3 (7.0–15.6) [*n* = 21]	9.6 (4.4–14.9) [*n* = 14]	0.413
Volume core infarct in mL, median (IQR)	19.33 (6.08–54.51)	15.68 (5.69–51.48)	25.15 (6.04–56.55)	0.501
Time from symptom onset to reperfusion ≤ 6h	17.26 (3.48–48.71) [*n* = 35]	18.05 (5.30–74.16) [*n* = 21]	5.97 (2.03–33.27) [*n* = 14]	0.281
Volume of peripheral infarcts in mL, median (IQR)	0.62 (0.12–1.57)	0.39 (0.10–1.62)	0.70 (0.21–1.55)	0.554
Time from symptom onset to reperfusion ≤ 6h	0.62 (0.10–1.53) [*n* = 35]	0.62 (0.10–1.65) [*n* = 21]	0.63 (0.08–1.01) [*n* = 14]	0.590

*significant (*p* < 0.05)

Two ANOVAs investigating the relationship of reperfusion and IV alteplase on number and volume of PE can be found in the supplemental material. We observed a significantly decreasing number of PE and by trend reduced volume of PE with higher reperfusion grades, while IV alteplase was neither associated with different number nor volume of PE for every reperfusion grade with significantly overlapping confidence intervals (*p* = 0.24; *p* = 0.65). (supplemental material).

Equivalence tests demonstrated that there is no association between the application of IV alteplase and the number or volume of PE. Based on a tolerated range of ± 1 PE number or ± 0.13 mL PE volume, respectively, equivalence may be assumed.

In univariable und multivariable ordinal regression analysis, there was no association between the application of alteplase and functional outcome (*p* > 0.1). Significant predictors of outcome were age (odds ratio for mRS shift: 1.06, *p* < 0.02) and ASPECTS (OR: 0.55, *p* = 0.001). Second, we investigated whether the volume or number of PE is associated with functional outcome. Neither number nor volume of peripheral emboli was significantly associated with the mRS at day 90 in this patient cohort (*p* = 0.95, *p* = 0.78). The volume of the DWI core lesion was significantly associated with outcome (odds ratio for mRS shift per mL: 1.02, 95% CI: 1.01–1.04, *p* < 0.001).

Finally, we analyzed the effect of intravenous alteplase on the functional outcome according to the baseline core lesion volume and investigated whether there is an interaction between intravenous lysis and core lesion volume. While core lesion volume was significantly associated with functional outcome (OR: 0.97, 95% CI: 0.95–0.99, *p* = 0.008; Supplemental Figure IV), there was no effect of intravenous lysis (*p* > 0.6), and the interaction term was not significant (*p* = 0.82).

## Discussion

Several randomized controlled trials are currently investigating the potential clinical benefit or harm of additional IV alteplase administration prior to thrombectomy (MR CLEAN- NO IV[15], SWIFT DIRECT, clinical trial ID: NCT03192332). Our data suggest that additional IV alteplase has no relevant impact on sub-angiographic peripheral DWI emboli in cases of successful reperfusion (eTICI ≥ 2b50).

Iatrogenic embolization attributed to MT represents a feared complication, potentially irreversibly compromising reperfusion success and patient outcome [16]. In a recent study, it has been observed that some of these emboli are not detectable on DSA but can be demarcated on follow-up MRI with high-resolution DWI imaging [10]. Depending on the volume, and perhaps more importantly the topography, these emboli could have a high impact on post-stroke outcome and cognitive recovery or decline [17, 18]. In the current debate concerning whether or not IV alteplase should be administered prior to MT, one of the main arguments for the additional pharmacological treatment is the possible improvement of microvascular reperfusion as well as lysis of distal emboli, generally seen as a potential complication following endovascular reperfusion [19]. Recently, the first randomized controlled trials on direct MT versus MT plus additional IV alteplase have been published showing that direct MT was non-inferior with regard to functional outcome [7]. In this context, the present study further corroborates these findings using a different metric. Investigating the influence of IV alteplase on sub-angiographic emboli, we neither observed differences in the prevalence of PE 24 h after endovascular treatment comparing patients with direct MT and those with additional IV alteplase, nor did we see a time-dependent effect in patients that received IV alteplase. The relevance of these findings is independent from the missing pre-procedural MRI studies, required to confirm the periprocedural nature of the PE. Although this missing data represents a limitation of this study, a positive effect of IV alteplase would be expected regardless if the origin of microemboli was pre-procedural or periprocedural [18]. Furthermore, the volume of PE in both groups with and without IV alteplase did not differ significantly. Consequently, additional IV alteplase neither led to reduced numbers nor did it reduce the size of the peripheral embolic lesions. Because our analysis only included cases with successful reperfusion (TICI ≥ 2b50), this finding suggests that additional IV alteplase has no strong effect on the smaller peripheral ischemic lesions following endovascular reperfusion. Likewise, the volume of core lesions did not differ significantly, confirming prior study results that the cumulative effect of IV lysis is likely marginal in cases with proximal LVO, thereby requiring a higher number needed to treat [19, 20]. In corroboration results of previous studies, there were no differences observed in the degree of successful reperfusion and thus, direct MT seems to be as effective as MT plus additional IV lysis [19]. The probability of a type II error, i.e., falsely missing a strong effect of IV alteplase on the incidence of peripheral emboli, was very low in our study. Nevertheless, we cannot conclude that IV alteplase is per se ineffective for LVO stroke since bridging therapy can in some cases lead to successful reperfusion before the patient even reaches an endovascular stroke center. Furthermore, a potential treatment effect of IV alteplase might lie beyond the observable volume of infarcted tissue [20].

In our cohort, the functional outcome at 90 days was comparably good between both groups. Accordingly, the clinical importance of microembolic ischemic lesions remains unclear. Their impact most likely lies outside the scope of the most commonly used global functional outcome measures like the mRS and could involve rather subtle differences in neurological dysfunction [21]. Further research could investigate whether PE have an effect on NIHSS scores stratified by symptoms, or with further neurological assessment scores that represent more subtle cognitive changes. Accordingly, Kaesmacher et al showed recently that infarcts larger than 2 cm distal to the occlusion in initially not the hypoperfused territory and new territories are associated with poor outcome [22]. Further studies are warranted to investigate the impact of interventional techniques and devices with regard to thrombus characteristics on the occurrence of PE after MT and their influence on outcome [16, 23]. Moreover, further studies might investigate the impact of intravenous alteplase on PE in patients without or failed endovascular recanalization, considering that the present study only analyzed patients with successful endovascular recanalization.

### Limitations

This prospective observational study is subject to all limitations associated with a non-randomized study design. The number of patients was relatively small due to strict inclusion criteria intending to obtain a homogenous patient cohort. Patients were required to undergo MRI examination without significant delay of diagnosis, or significant logistic efforts. Hence, patients who have showed significant clinical complications such as severe and/or sudden neurological deterioration leading to ineligibility of MR imaging, or requiring rapid imaging to exclude severe secondary parenchymal hemorrhage, could not be included in this study, which potentially affects patients who suffered from symptomatic intracranial hemorrhage. Furthermore, since MRI imaging was not a part of the initial stroke imaging protocol for suspected LVO stroke, evidence is missing to confirm the periprocedural nature of all PE. Hence, patients receiving both IV lysis plus MT could have presented with an already higher number/volume of PE at baseline, which affects the evaluation of a protective effect of IV lysis in the follow-up imaging. However, it is important to point out that the patient groups did not show any significant differences in baseline and imaging parameters including atrial fibrillation. Second, a sensitive detection of PE in baseline imaging is not feasible, due to the long acquisition time of high-quality/high-resolution DWI (7.6 min in the present study). Although shorter high-resolution DWI sequences have been tested, these may not have sufficient resolution to detect and quantify small PE. In this cohort, 53% of PE apparent on high-resolution DWI were missed in conventional DWI. While the utilization of MRI for acute stroke triage may have significant advantages (e.g., unknown onset stroke) and is used as a primary modality in several stroke centers [24, 25], CT is still the most widely performed initial imaging modality to detect LVO stroke, due to its applicability and availability [3, 8]. Although MRI was reported to be beneficial to guide IV lysis in WAKE-UP stroke patients, the positive effect of IV lysis on functional outcome in patients included in the WAKE-UP trial with an initial NIHSS > 10, as observed in the majority of proximal LVO stroke, was unclear [26, 27]. Representing a real-world LVO patient cohort, the median NIHSS in this study was 14 (IQR: 10–17), and thus, the general use of MRI would have had highly affected general and personal clinical equipoise to include patients in this study [28].

### Conclusion

Our study does not confirm the hypothesis that the additional administration of IV alteplase prior to mechanical thrombectomy leads to a significant post-procedural reduction in the number or volume of peripheral microembolic lesions visible on high-resolution DWI. Further studies are needed to investigate potential influencing factors of microemboli after thrombectomy, as well as their clinical impact.

## Supplementary information

ESM 1 (DOCX 841 kb)

## Abbreviations

ASPECTS: Alberta Stroke Program Early CT Score
CI: Confidence interval
DSA: Digital subtraction angiography
DWI: Diffusion-weighted imaging
eTICI: Expanded thrombolysis in cerebral infarction
IQR: Interquartile range
LVO: Large vessel occlusion
MRI: Magnetic resonance imaging
mRS: Modified Rankin scale
MT: Mechanical thrombectomy
NIHSS: National Institute of Health Stroke Scale
PE: Peripheral emboli

## Declarations

### Guarantor

The scientific guarantor of this publication is Gabriel Broocks.

### Statistics and biometry

Gerhard Schön kindly provided statistical advice for this manuscript.

### Methodology

• prospective

• observational

• performed at one institution
