# Supplementary figures and images for: Impact of intravenous alteplase on sub-angiographic emboli in high-resolution diffusion-weighted imaging following successful thrombectomy
Source: Eur Radiol. 2021 May 8;31(11):8228–35. doi: 10.1007/s00330-021-07980-0 (PMC8523452; doi:10.1007/s00330-021-07980-0)

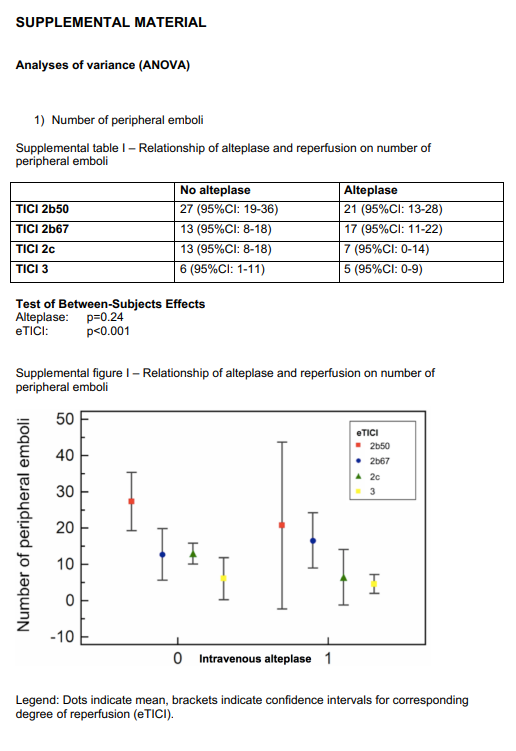


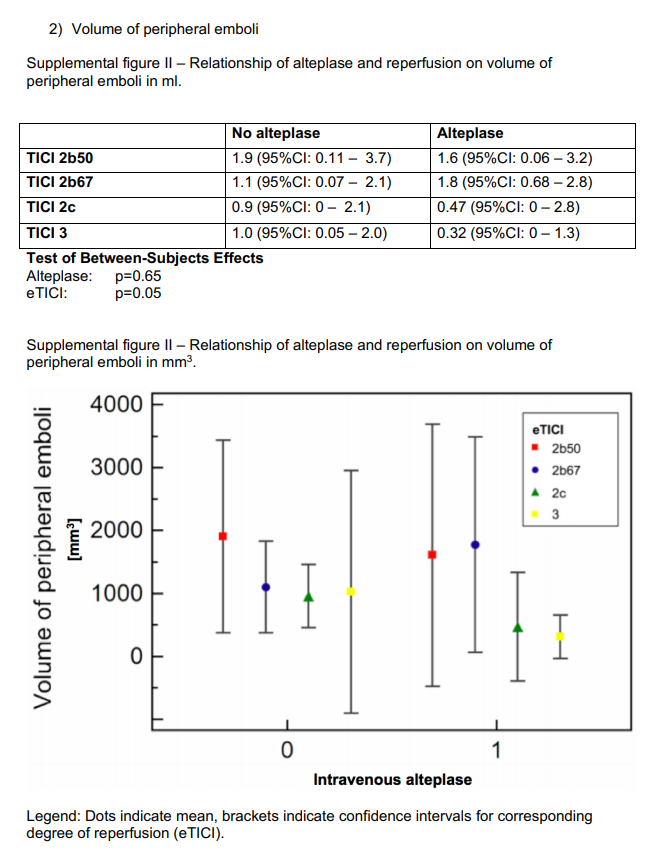


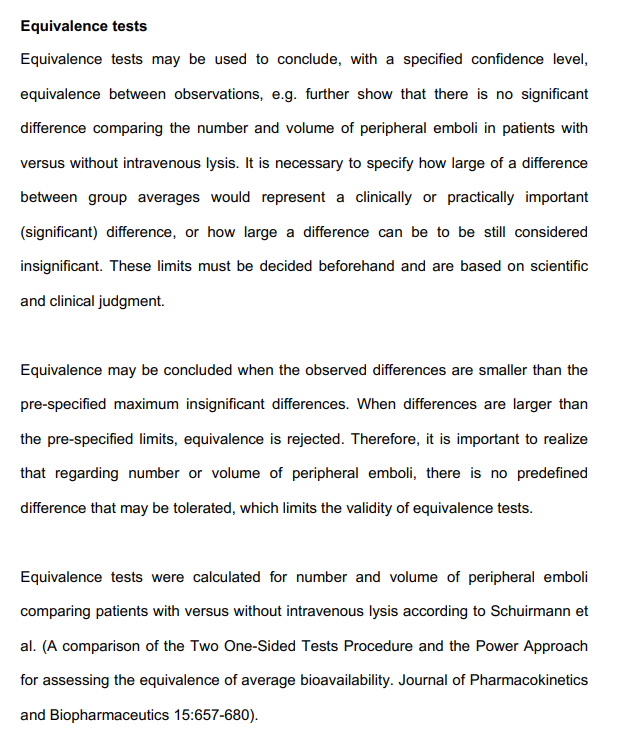


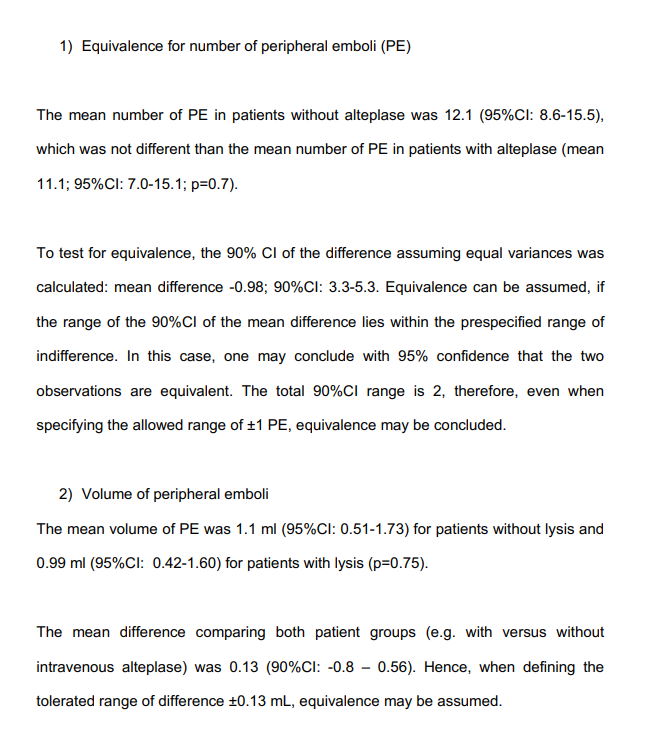


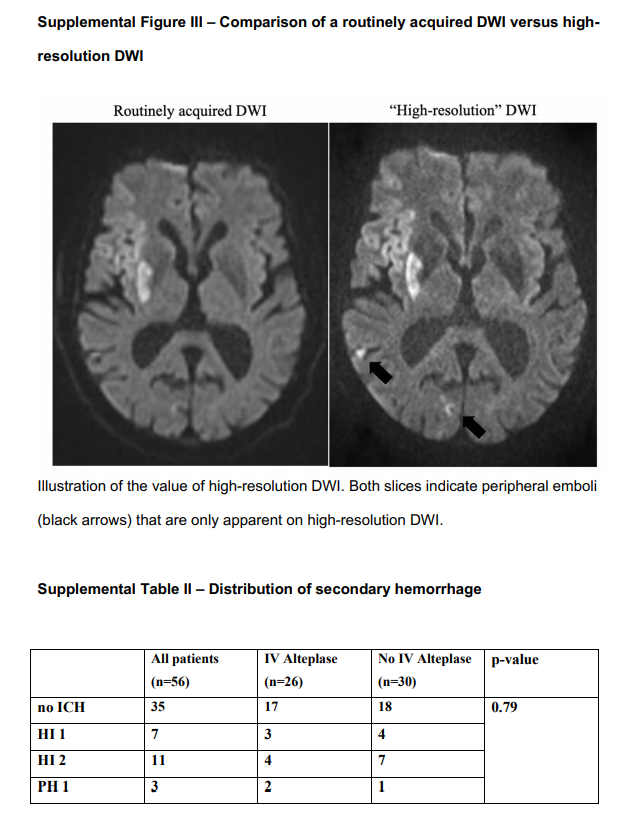


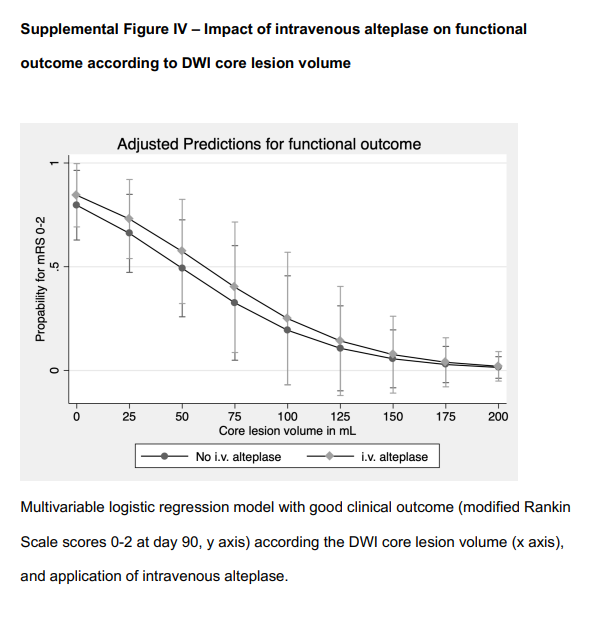


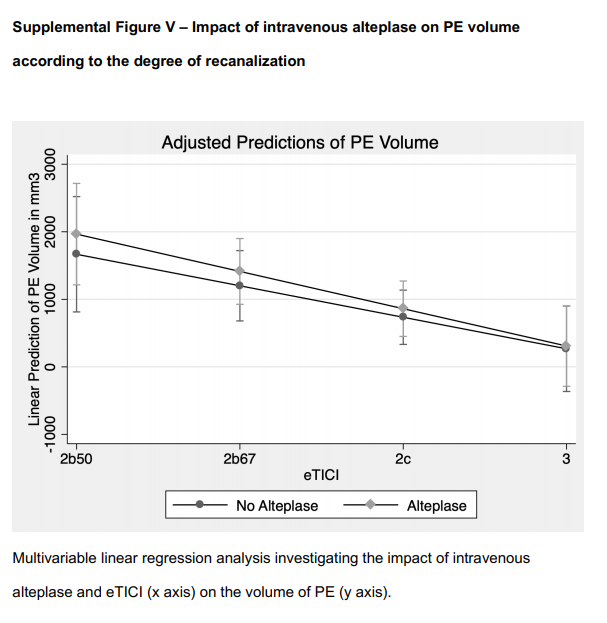

Supplement: Supplementary file 1 — (DOCX 841 kb) [file 330_2021_7980_MOESM1_ESM.docx]
